# Association of Sasang Constitutional Type with Sarcopenia

**DOI:** 10.1155/2015/651090

**Published:** 2015-11-12

**Authors:** Seung Ku Lee, Dae Wui Yoon, Kyung-Mee Choi, Si Woo Lee, Jong Yeol Kim, Jin Kwan Kim, Chol Shin

**Affiliations:** ^1^Institute of Human Genomic Study, College of Medicine, Korea University Ansan Hospital, 516 Gojan-1-dong, Danwon-gu, Gyeonggi-do, Ansan 425-707, Republic of Korea; ^2^Constitutional Medicine and Diagnosis Research Group, Korea Institute of Oriental Medicine, 461-24 Jeonmin-dong, Yuseong-gu, Daejeon 305-811, Republic of Korea; ^3^Medical Research Division, Korea Institute of Oriental Medicine, 461-24 Jeonmin-dong, Yuseong-gu, Daejeon 305-811, Republic of Korea; ^4^Department of Biomedical Laboratory Science, Jungwon University, 85 Munmu-ro, Goesan-eup, Goesan-gun, Chungbuk, Republic of Korea; ^5^Department of Pulmonary, Sleep and Critical Care Medicine, College of Medicine, Korea University Ansan Hospital, 516 Gojan-1-dong, Danwon-gu, Gyeonggi-do, Ansan 425-707, Republic of Korea

## Abstract

Sasang constitutional medicine (SCM) is a unique Korean traditional medicine that classifies human beings as four distinct types named Sasang constitutional types (SCTs), based on physiologic, physical, and psychological traits. Accumulating evidence has demonstrated that specific constitutional types are associated with chronic diseases, but no study has investigated the relationship between SCTs and sarcopenia. The aim of this study was to examine the association in a large population-based study. Data from 1,204 participants who completed questionnaires for life style, anthropometric evaluation, and biochemical analysis were analyzed. Classification of the SCTs was done using an integrated diagnostic method. Sarcopenia was defined as appendicular skeletal muscle mass/height^2^ less than one standard deviation below the gender-specific normal mean of a younger group. Dual-energy X-ray absorptiometry was used to assess whole body composition. The prevalence of sarcopenia was 8.6% in the Tae-eum (TE) type, 44.7% in the So-eum (SE) type, and 20.7% in the So-yang (SY) type. Multivariate analysis revealed that the SE and SY types had 9.22 (5.06–16.81; *P* < 0.0001) and 2.90 (1.76–4.76; *P* < 0.0001) greater odds of sarcopenia compared to the TE type, respectively. Our results show that the SE and SY types are significantly associated with increased prevalence of sarcopenia.

## 1. Introduction

Sasang constitutional medicine (SCM), a unique traditional medicine long used to diagnose and treat patients in Korea, originated from the Korean physician Jema Lee (AD 1837–1900) in 1894. SCM classifies human beings into four Sasang constitutional types (SCTs), namely, Tae-yang (TY), Tae-eum (TE), So-yang (SY), and So-eum (SE), based on distinct physiological, physical, and emotional characteristics, drug response, and susceptibility to specific diseases [1]. Such classifications have been employed for prediction of specific vulnerability to some diseases, as well as for prescription of herbal medicine in a way that minimizes side effects. The concept of SCM corresponds to the personalized or tailored approach of modern medicine. Even though it has been used in Korea for over 120 years, the lack of objective data gathered based on modern technology and scientific knowledge to support the SCM theory makes many doctors hesitant to apply it to diagnosis or treatment of their patients. Researchers have sought scientific evidence to support relationships between SCTs and specific diseases or symptoms, and it has been demonstrated that some types are significantly and independently associated with chronic and metabolic diseases such as hypertension (HTN), diabetes mellitus (DM), obesity, and metabolic syndrome [2–4].

Sarcopenia is a syndrome first proposed by Rosenberg to describe age-related decreases of muscle mass [5]. It has been defined by a progressive and generalized decrease of skeletal muscle mass and strength occurring with advancing age [6] that is caused by numerous factors including a lack of physical activity, changes in protein metabolism, alterations in endocrine hormones, elevation of proinflammatory cytokines, and apoptosis [7–9]. Many studies have shown that a decline in muscle mass and function occurs not only with aging, but also in relatively healthy elderly people [10, 11]. Sarcopenia is associated with an increased risk of osteoporosis and fractures in aging populations, as well as poor quality of life and increased mortality [7, 12, 13]. All of these conditions are associated with increasing socioeconomic costs [14].

In Korea, the elderly population grew significantly by 24.3% between 2005 and 2010 [15], attracting attention to care for physical and social functioning and quality of life in elders. The prevalence of sarcopenia increases with advancing age, and an appropriate classification or prediction of individuals who have a high risk of sarcopenia could help reduce the associated socioeconomic cost, especially in elderly people. To our knowledge, however, no study has classified a high risk group for sarcopenia in terms of SCM. Thus, we tried to investigate whether a specific type of constitution was independently associated with the risk of sarcopenia using data from a large population-based cohort in Korea.

## 2. Materials and Methods

### 2.1. Study Population

Data from subjects enrolled in the Korean Genome and Epidemiology Study (KoGES), which is an ongoing population-based study, were employed for the analysis. We selected the “Ansan cohort” of 5,012 volunteers aged 40–69 years, based in an urban area and conducted at Korea University Ansan Hospital starting in 2001. The participants were followed biennially with a scheduled site visit for interviews and health examinations for anthropometric measurements and blood biochemical analyses. They also completed an interviewer-administered questionnaire on demographic information, medical history, and health conditions. In this study, analyses were conducted on 1,204 participants (600 men, 604 women) who were enrolled between 2009 and 2011 and had completed data available on the questionnaire, anthropometric evaluation, and dual-energy X-ray absorptiometry (DXA). All participants provided written informed consent. The study protocol was approved by the Human Subjects Review Committee at Korea University Ansan Hospital.

### 2.2. Classification of SCTs

In order to classify each type of Sasang constitution, an integrated diagnostic method was used [16]. The diagnostic method is based on a holistic approach using integrated values of individual quantitative data including objectively measured facial features, body shape, voice, and questionnaire responses. Briefly, facial features were acquired from photographs with a digital camera followed by image processing. Variables for body shape analysis included circumferences of eight parts, ratios of pairs of the circumferences, height, weight, and body mass index (BMI). Voice parameter analysis was performed using the Hidden Markov Model Toolkit (Cambridge University Engineering Department, Cambridge, UK) and the Pratt voice analysis program (University of Amsterdam, Amsterdam, Netherlands). The questionnaire consisted of 67 multiple-choice questions to assess participant temperaments and specific physiological symptoms. Since no one was classified as the TY type, only data from the TE, SE, and SY constitutions were evaluated in this study.

### 2.3. Demographic, Anthropometric, and Laboratory Measurements

Weight was measured to the nearest 0.1 kg using an electronic scale. Monthly income was categorized as ≤2,000 US dollar (USD), 2,000–4,000 USD, and >4,000 USD. Blood pressure (BP) was measured by trained examiners using an appropriately sized cuff and a mercury sphygmomanometer according to a standardized protocol. The use of antihypertensive and antihyperglycemic medications and diagnosis was assessed by an interviewer-administered questionnaire at visits. HTN was defined if the participants had systolic BP ≥ 140 mmHg, had diastolic BP ≥ 90 mmHg, or were being treated with antihypertensive medication. DM was defined when fasting glucose exceeded 126 mg/dL or antihyperglycemic agents were used. BMI was calculated as weight in kilograms divided by the square of the height in meters (kg/m^2^). We calculated beverage-specific alcohol consumption in g/d on the basis of the alcohol content (4.5% for beer, 13% for wine, 40% for hard liquor, 22% for soju, 15% for chungju, and 6% for makgeolli), the frequency of drinking, and the amount consumed. Leisure-time physical activity was evaluated using questionnaires covering the type of activity, frequency, and duration. A metabolic equivalents (MET) score was calculated by multiplying hours spent by MET values. Biochemical analyses were conducted to determine serum levels of fasting insulin and glucose, high density lipoprotein (HDL) cholesterol, and triglycerides (TG) using an autoanalyzer (ADVIA 1650, Siemens, Tarrytown, NY, USA) in the Seoul Clinical Laboratories (Seoul, Korea).

### 2.4. Dual-Energy X-Ray Absorptiometry and Definitions of Sarcopenia

Whole body composition was assessed using dual-energy X-ray absorptiometry (DXA) (DPX-MD+, General Electric, Madison, WI, USA). Appendicular skeletal muscle mass (ASM) was defined as the sum of the lean soft tissue masses for the arms and legs, assuming that all nonfat and nonbone tissue was skeletal muscle. Sarcopenia was defined as an ASM/height^2^ less than 1 standard deviation (SD) below the gender-specific normal mean of a younger reference group of our population (class 1 sarcopenia) (men and women aged 47–50 yrs; *n* = 517) [17]. The cutoff values for sarcopenia based on the 1 SD below the mean of young adults in this study were 7.34 kg/m^2^ and 5.65 kg/m^2^ for men and women, respectively. Class 2 sarcopenia, defined as an ASM/height^2^ less than 2 SD below the gender-specific normal mean of young adults (cutoff value 6.60 kg/m^2^ for men and 5.10 kg/m^2^ for women), was not considered in our study since such criteria resulted in a very low frequency of sarcopenia in total men (*n* = 18, 3%) and women (*n* = 13, 2.2%).

### 2.5. Statistical Analysis

Demographic variables between SCTs were expressed as mean ± SD. Differences of the means were evaluated using one-way analysis of variance (ANOVA) for continuous variables and chi-square test for categorical variables. Bonferroni's* post hoc* test was performed for multiple comparisons. In addition, we conducted a multiple logistic regression analysis to evaluate an odds ratio (OR) for the presence of sarcopenia in relation to different SCTs with a 95% confidence interval (CI) and *P* value. Potential confounding variables adjusted for the multivariate logistic regression analysis were age, sex, waist, smoking, total fat mass, alcohol consumption, physical activity, monthly income, and presence of HTN. We repeated a multiple logistic regression stratified by gender to identify an independent association. Statistical analysis was done using SAS version 9.3 (SAS Institute, Cary, NC, USA). All *P* values < 0.05 were considered statistically significant.

## 3. Results

### 3.1. Characteristics of the Study Population

The characteristics of study subjects are presented in Table 1. There was a significant gender imbalance in distribution among the groups. The TE type was older, heavier, and taller and consumed more alcohol than the SE and SY types. No significant difference was observed in physical activity among the SCTs. There were significant differences in the smoking status and monthly income among the SCTs. Presence of HTN and DM and fasting insulin level were the highest in the TE type. The TE type had the highest values of all metabolic parameters except for HDL cholesterol.

### 3.2. Body Composition and Prevalence of Sarcopenia according to SCTs

Total body fat, total body fat mass, total lean body mass, total body mineral density, and ASM/height^2^ were all highest in the TE type. The SE type had the lowest total body fat, total body fat mass, total body mineral density, and ASM/height^2^. Only total lean body mass was lowest in the SY type. When a cutoff point of one SD for ASM/height^2^ in the young reference group was used to define sarcopenia, the prevalence of sarcopenia was 8.6% in the TE type, 44.7% in the SE type, and 20.7% in the SY type (Table 2).

### 3.3. Odds Ratios for Sarcopenia according to SCTs

The association of sarcopenia and SCT is shown in Table 3. In a crude logistic regression analysis conducted in both sexes, the SE type had 8.98-fold (95% CI, 5.88–13.72; *P* < 0.0001) higher odds of having sarcopenia compared to the TE type. The SY type had 2.91-fold (95% CI, 1.99–4.25; *P* < 0.0001) greater odds of having sarcopenia. In the multivariate analysis adjusted for age, sex, waist, total fat mass, alcohol consumption, physical activity, monthly income, smoking, and presence of HTN, the odds for sarcopenia in the SE type increased to 9.22-fold (95% CI, 5.06–16.81; *P* < 0.0001) whereas that of the SY type showed no remarkable change (OR = 2.90; 95% CI, 1.76–4.76; *P* < 0.0001). In a gender-specific analysis, men who were classified as SE type had higher odds of having sarcopenia compared to the TE type in both the crude (OR = 8.67; 95% CI, 4.98–15.09; *P* < 0.0001) and multivariate analysis (OR = 16.51; 95% CI, 6.81–39.99; *P* < 0.0001). Men in the SY type also had greater odds of having sarcopenia in both the crude (OR = 2.30; 95% CI, 1.36–3.91; *P* = 0.002) and multivariate analysis (OR = 2.72; 95% CI, 1.35–5.48; *P* = 0.0051). Women who belonged to the SE type had 10.13-fold greater odds of having sarcopenia than the TE type (95% CI, 5.14–19.96; *P* < 0.0001) in the crude analysis but the odds decreased to 7.25 in the multivariate analysis (95% CI, 2.99–17.57; *P* < 0.0001). Women classified as SY type had 3.77 times higher odds of sarcopenia than the SE type (95% CI, 2.09–6.79; *P* < 0.0001), but the ORs decreased in multivariate analysis to 3.12 (95% CI, 1.46–6.67; *P* = 0.0034).

## 4. Discussion

In this population-based study, we found that SE and SY type were significantly associated with increased prevalence of sarcopenia, even after adjusting for potential confounders, but the odds of having sarcopenia were much higher in the SE type than the SY type. Moreover, men who belonged to the SE type had higher odds of having sarcopenia than women in the SE type, but this trend was reversed for the SY type.

The prevalence of sarcopenia varies according to region, age, definitions, and measuring techniques. In a systematic review of the prevalence of sarcopenia in Asian and Caucasian adults aged more than 50 years using the definition proposed by the European Working Group on Sarcopenia in Older People, the prevalence was 1–29% in community-based populations and 14–33% in long-term care populations [18].

Sarcopenia is significantly associated with physical dysfunctioning and several diseases. In a study using data from the Third National Health and Nutrition Examination Survey, the risk of having functional impairment and disability was approximately twofold higher in groups with sarcopenia than those without [19]. There was a negative association of sarcopenia with the third quartile and fourth quartile of walking physical activity in elderly Korean men aged more than 60 years [20]. Sarcopenia significantly increased the risk of osteoporosis about 4.1 times in men and 1.9 times in women compared to normal groups [15]. Sarcopenia has been associated with increased mortality in several studies. In a study using data from 1,396 community-dwelling participants aged 70 years, even after controlling for possible confounders, low muscle mass was significantly associated with increased mortality (hazard ratio 1.95; 95% CI, 1.25–2.00) [21]. A prospective study that followed 715 older men revealed that loss of appendicular skeletal muscle mass was associated with a 1.61-fold increased risk of all-cause mortality per 1-SD decrease [22].

Because there are several ways and definitions to define sarcopenia, the prevalence can vary depending on the method used. Several techniques for measuring sarcopenia such as anthropometric measurement, DXA, bioelectrical impedance, computed tomography, and magnetic resonance imaging (MRI) exist. Among them, MRI is the most accurate technique, but its high cost limits application in a large population-based study. DXA is the most frequently used method to measure muscle mass due to its high correlation with MRI, accuracy, reliability, and minimal radiation exposure [23]. Within DXA studies, appendicular muscle mass index (AAMI), defined as appendicular muscle mass divided by the square of the height in meters, is widely used with combinations of different criteria such as two SD below the mean of a young reference group, or the 20th percentile below the elderly sample distribution, residual method, and ROC curve analysis. Baumgartner et al. reported the prevalence of sarcopenia in the US as 28.5% and 33.9% in men and women over 60 years old, respectively, using an AAMI of two SD below the gender-specific mean of young adults (18–40 yrs) [24]. Asian studies report a lower prevalence of sarcopenia than those in western countries. In China, the prevalence of sarcopenia was 2.2–7.1% in men and 2.6–6.1% in women when sarcopenia was defined as an AAMI of 2 SD below the gender-specific mean of young adults (20–40 yrs) [25, 26]. In Korea, the prevalence of sarcopenia in subjects of more than 60 yrs was 6.3% in men and 4.1% in women when using the same definition as the study conducted in China [27].

Pathogenesis of sarcopenia is multifactorial, with no single cause or underlying mechanism. Physical inactivity, insufficient intake of protein, decrease in growth hormone and testosterone, increase in cortisol and proinflammatory cytokines, and apoptosis are regarded as contributing factors for the development of sarcopenia [9]. The reason why the SE type has a much higher prevalence of sarcopenia than other types is unknown, but ineffective processing of raw materials could be one of the causes. According to SCM theory, the SE type has a hypoactive spleen, which results in poor management of food intake and digestion. Supporting the hypothesis, a higher frequency of digestive system dysfunction such as chronic indigestion and gastroptosis has been reported in the SE type compared to the other constitutional types [28, 29]. This observation implies that even when the same amount of amino acids for muscle protein synthesis is supplied, SE individuals are relatively insufficient in using amino acids compared to other types. Dietary information, including amount of protein intake, was not examined in our study and may provide supporting evidence for the hypothesis. A further study is required to elucidate causality.

Many studies report no significant association of gender with sarcopenia prevalence, but findings are not conclusive [30–32]. Interestingly, we observed different odds of having sarcopenia according to gender in the SY and SE types. Women who were classified as the SY type had greater odds of having sarcopenia than men in both the crude and multivariate analyses, while the odds of sarcopenia were lower in SE type women than SE type men in the multivariate analysis, implying the presence of different gender-specific contributing factors to sarcopenia according to the SCTs.

The main strengths of this study were the large number of participants and the general population-based sample. However, several limitations should be recognized. First, the number of participants who belong to the SE type was relatively small compared to the other types. Second, we did not have dietary information on nutritional status for energy intake or protein intake. Third, we did not measure muscle strength in the participants, which is known as a better predictive factor for mobility, physical performance, and mortality than muscle mass [33–35]. Thus, whether the SE and SY type actually suffer more from adverse outcomes such as functional impairment and physical disability than the TE type is unknown. Fourth, age distributions of the participants were limited to middle age; thus our results cannot be extrapolated to sarcopenic features of all age groups.

## 5. Conclusions

SE and SY type are significantly associated with increased prevalence of sarcopenia. These results provide evidence that classification by SCTs can be used for early prevention in individuals who are vulnerable to sarcopenia.

## Figures and Tables

**Table 1:** General characteristics of participants according to Sasang constitutional type.

	TE	SE	SY	*P* value
Participants, *n* (women %)	638 (40.6)	161 (50.9)	405 (64.9)	<0.0001
Age (y)	57.2 ± 7.7^de^	54.1 ± 5.6	54.9 ± 6.3	<0.0001
BMI (kg/m^2^)	26.5 ± 2.3^de^	21.5 ± 1.6	23.1 ± 1.9^d^	<0.0001
Weight (kg)	70.3 ± 8.6^de^	55.7 ± 6.1	58.3 ± 7.3^b^	<0.0001
Height (cm)	162.9 ± 8.3^ae^	160.9 ± 7.1	158.8 ± 7.9^a^	<0.0001
Physical activity (MET-hours/day)	203.4 ± 266.9	162.8 ± 216.5	210.1 ± 299.2	0.1603
Alcohol consumption (g/day)	11.0 ± 20.1^ae^	6.7 ± 22.9	5.0 ± 12.4^d^	<0.0001
Fasting insulin (*μ*IU/mL)	10.5 ± 9.0^de^	7.2 ± 2.8	8.0 ± 3.4^d^	<0.0001
Smoker, *n* (%)	93 (14.6%)	24 (14.9%)	39 (9.6%)	0.0497
Monthly income, *n* (%)				
≤2000 USD	188 (29.5%)	31 (19.3%)	107 (26.4%)	
2000–4000 USD	254 (39.8%)	81 (50.3%)	179 (44.2%)	0.0132
>4000 USD	196 (30.7%)	49 (30.4%)	119 (29.4%)	0.2504
HTN case, *n* (%)	272 (42.6%)^de^	30 (18.6%)	77 (19.0%)	<0.0001
DM case, *n* (%)	144 (22.6%)^de^	11 (6.8%)	46 (11.4%)	<0.0001
Anti-HTN drug, *n* (%)	200 (31.4%)^de^	21 (13.0%)	52 (12.8%)	<0.0001
Anti-DM drug, *n* (%)	71 (11.1%)^bc^	5 (3.1%)	19 (4.7%)	<0.0001
Metabolic parameters				
Waist (cm)	85.9 ± 6.3^de^	73.4 ± 5.5	75.8 ± 5.9^d^	<0.0001
SBP (mmHg)	117.9 ± 13.9^de^	111.1 ± 13.3	111.8 ± 15.1^d^	<0.0001
DBP (mmHg)	77.8 ± 9.3^ce^	74.5 ± 8.5	74.4 ± 9.1^d^	<0.0001
HDL (mg/dL)	42.3 ± 9.1^de^	48.3 ± 11.9	46.5 ± 11.0^d^	<0.0001
TG (mg/dL)	163.2 ± 121.9^de^	118.6 ± 60.4	128.2 ± 80.7^d^	<0.0001
Fasting glucose (mg/dL)	103.3 ± 25.3^de^	93.0 ± 16.3	95.5 ± 23.0^d^	<0.0001

Values are expressed as mean ± standard deviation.

TE: Tae-eum type; SE: So-eum type; SY: So-yang type; BMI: body mass index; MET: metabolic equivalents per hour daily; HTN: hypertension; DM: diabetes mellitus; SBP: systolic blood pressure; DBP: diastolic blood pressure; HDL: high-density lipoprotein; TG: triglyceride.

^a^ *P* < 0.05 versus SE type.

^b^ *P* < 0.01 versus SE type.

^c^ *P* < 0.001 versus SE type.

^d^ *P* < 0.0001 versus SE type.

^e^ *P* < 0.0001 versus SY type.

**Table 2:** Difference of body composition from dual-energy X-ray absorptiometry according to Sasang constitutional type.

	TE	SE	SY	*P* value
Total body fat (kg)	66.5 ± 8.4^a^	52.4 ± 6.0	54.9 ± 7.3^b^	<0.0001
Total body fat mass (kg)	20.8 ± 5.6^a^	12.9 ± 4.9	15.9 ± 4.7	<0.0001
Total lean body mass (kg)	45.7 ± 8.6^a^	39.5 ± 7.0	39.1 ± 8.2	<0.0001
Total body mineral density (kg)	2.7 ± 0.5^a^	2.35 ± 0.4	2.4 ± 0.4	<0.0001
ASM/height^2^	7.52 ± 1.08^a^	6.55 ± 0.96	6.64 ± 1.02	<0.0001
Sarcopenia, *n* (%)	55 (8.6%)	72 (44.7%)	84 (20.7%)	<0.0001

Sarcopenia was defined as an ASM/height^2^ less than 1 SD below the gender-specific normal mean of a younger reference group of our population.

The cutoff values were 7.34 kg/m^2^ and 5.65 kg/m^2^ for men and women, respectively.

TE: Tae-eum type; SE: So-eum type; SY: So-yang type; ASM: appendicular skeletal muscle mass.

^a^ *P* < 0.0001 versus SE and SY type.

^b^ *P* < 0.01 versus SE type.

**Table 3:** Odds ratio of sarcopenia in relation to Sasang constitutional type.

		Normal, *n* (%)	Sarcopenia, *n* (%)	Crude OR (95% CI), *P* value	Multivariate OR (95% CI), *P* value^a^
Total	TE	583 (91.4%)	55 (8.6%)	Reference	Reference
	SE	89 (55.3%)	72 (44.7%)	8.98 (5.88–13.72), <0.0001	9.22 (5.06–16.81), <0.0001
	SY	321 (79.3%)	84 (20.7%)	2.91 (1.99–4.25), <0.0001	2.90 (1.76–4.76), <0.0001

Men	TE	341 (90.0%)	38 (10.0%)	Reference	Reference
	SE	40 (50.6%)	39 (49.4%)	8.67 (4.98–15.09), <0.0001	16.51 (6.81–39.99), <0.0001
	SY	112 (78.9%)	30 (21.1%)	2.30 (1.36–3.91), 0.002	2.72 (1.35–5.48), 0.0051

Women	TE	242 (93.4%)	17 (6.6%)	Reference	Reference
	SE	49 (59.8%)	33 (40.2%)	10.13 (5.14–19.96), <0.0001	7.25 (2.99–17.57), <0.0001
	SY	209 (79.5%)	54 (20.5%)	3.77 (2.09–6.79), <0.0001	3.12 (1.46–6.67), 0.0034

^a^Data were adjusted for age, sex, waist (cm), total fat (kg), alcohol consumption (g/d), physical activity (metabolic equivalent per hour daily), monthly income (≤2,000 USD, 2,000–4,000 USD, and >4,000 USD), smoking, and presence of hypertension.

TE: Tae-eum type; SE: So-eum type; SY: So-yang type; OR: odds ratio.
